# Enhanced transfection of cell lines from Atlantic salmon through nucoleofection and antibiotic selection

**DOI:** 10.1186/1756-0500-4-136

**Published:** 2011-05-06

**Authors:** Berit L Schiøtz, Esther G Rosado, Espen S Baekkevold, Morten Lukacs, Siri Mjaaland, Hilde Sindre, Unni Grimholt, Tor Gjøen

**Affiliations:** 1 Department of Pharmaceutical Biosciences, School of Pharmacy, University of Oslo, Norway; 2 Department of Microbiology, Faculty of Science, University of Malaga, Malaga, Spain; 3 Institute of Pathology, Rikshospitalet-Radiumhospitalet Medical Center, University of Oslo, Norway; 4 Department of Basic Science and Aquatic Medicine, Norwegian School of Veterinary Science, Oslo, Norway; 5 Department of Food Safety and Infection Biology, Norwegian School of Veterinary Science, Oslo, Norway; 6 Department of Bacteriology and Immunology, The Norwegian Institute of Public Health, P.O. Box 4404 Nydalen, N-0403 Oslo, Norway; 7 National Veterinary Institute, Oslo, Norway; 8 Centre for Ecology and Evolutionary Synthesis, Department of Biology, University of Oslo, Norway

## Abstract

**Background:**

Cell lines from Atlantic salmon kidney have made it possible to culture and study infectious salmon anemia virus (ISAV), an aquatic orthomyxovirus affecting farmed Atlantic salmon. However, transfection of these cells using calcium phosphate precipitation or lipid-based reagents shows very low transfection efficiency. The Amaxa Nucleofector technology™ is an electroporation technique that has been shown to be efficient for gene transfer into primary cells and hard to transfect cell lines.

**Findings:**

Here we demonstrate, enhanced transfection of the head kidney cell line, TO, from Atlantic salmon using nucleofection and subsequent flow cytometry. Depending on the plasmid promoter, TO cells could be transfected transiently with an efficiency ranging from 11.6% to 90.8% with good viability, using Amaxa's cell line nucleofector solution T and program T-20. A kill curve was performed to investigate the most potent antibiotic for selection of transformed cells, and we found that blasticidin and puromycin were the most efficient for selection of TO cells.

**Conclusions:**

The results show that nucleofection is an efficient way of gene transfer into Atlantic salmon cells and that stably transfected cells can be selected with blasticidin or puromycin.

## Findings

Introduction of nucleic acids into cells by non-viral methods, transfection, has been an important tool in many aspects of cell and molecular biology since its introduction more than 30 years ago [1,2]. Transfection of cells with plasmids encoding a gene of interest coupled to a reporter gene, e.g. green fluorescent protein (GFP) has become a pivotal technique for the study of gene expression, protein trafficking and localization [3]. Since these pioneering studies, generation of pure plasmid DNA has become a routine task and other methods for introduction and expression of foreign nucleic acids in cells have been developed. These methods have later been optimized mainly for use with mammalian cell lines, and with the right combination of cell type and method, almost 100% transfection efficiency can be achieved [4]. However, when applying the same methods to cells from other vertebrates like fish cultured at lower temperatures (5-15°C), the efficiency is often below 10% [5-7]. For analysis of the expressed gene product by microscopy this can be sufficient, but for biochemical studies or applications like siRNA, a higher transfection efficiency is desired. When plasmids are introduced for the purpose of generating recombinant viruses, high transfection efficiency is also critical for successful rescue. Cell lines from various fish species have been successfully used for both stable transfection and rescue of recombinant viruses [8,9]. In two papers, promoter optimization and selection of stable cell lines from crucian carp was reported [6,10]. Others have reported generation of enhanced green fluorescent protein (EGFP) or MX expressing cell lines (CHSE 214) from Chinook salmon [11,12]. In addition there are several reports on successful expression of viral glycoproteins (DNA vaccination) in fish, in vivo [13]. However, although transgenes have been transiently expressed in Atlantic salmon cells both *in vivo *[14] and *in vitro *[15,16], the generation of stably expressing cell lines has not been reported for cells from Atlantic salmon. Given the importance of this species in fish research and aquaculture we have investigated methods for transfection, expression and selection of transformed cells from this species. Here we describe a transfection method for Atlantic salmon cell lines using the Nucleofector technology. The principle of nucleofection is that a combination of electrical parameters and cell-type specific nucleofection solutions ensures efficient delivery of DNA to the nucleus, combined with low toxicity and high cell viability. We have used 3 different cell lines from Atlantic salmon: SHK-1, TO and ASK. They all originate from long term cultures of head kidney cells (mainly leucocytes) but show different expression profiles [17] and vary in their ability to propagate and diagnose infectious salmon anemia virus [18,19].

### Amaxa electroporation

**Figure 1:**
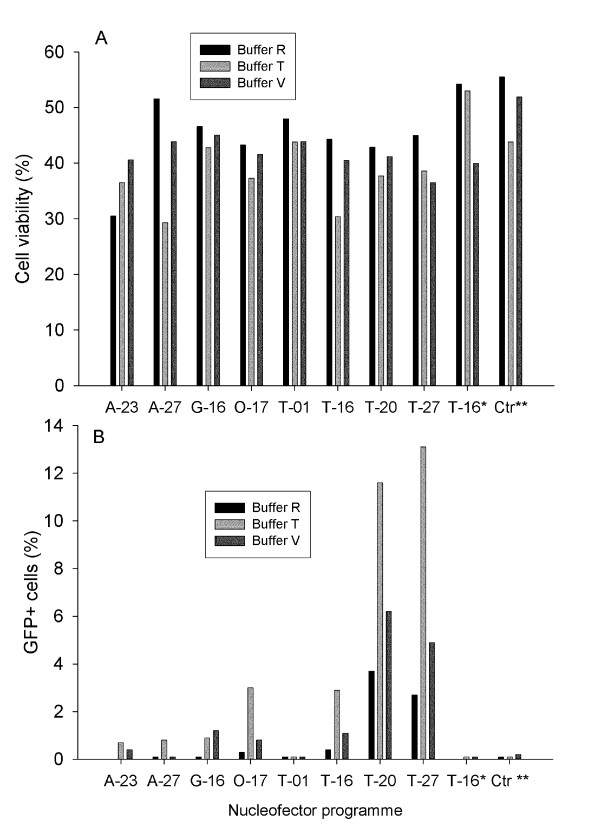
**Cell viability and transfection efficiency after nucleofection of TO cells**. A total of 5 × 10^6 ^cells were transfected with 5 μg pFRT/lacZeo Flp, using buffer R, T or V, and a range of programs. Three days after transfection, cell viability (a) and GFP expression (b) was quantified by flow cytometry. * = Electroporation, no plasmid ** = Plasmid, no electroporation program. Data from a representative experiment (out of 3) is depicted here.

**Figure 2:**
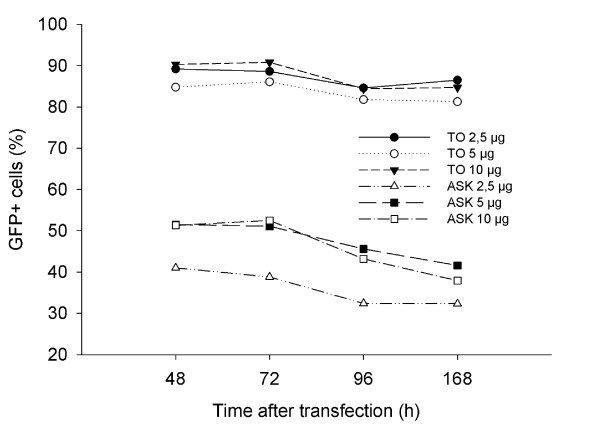
**Effect of DNA concentration on nucleofection frequency**. GFP expression was analyzed by flow cytometry 48, 72, 96 and 168h after transfection of TO or ASK cells with varying amounts (2.5, 5 or 10 μg) of pmaxFP-Green-N. Data from a representative experiment (out of 3) is depicted here.

The methods used are described in additional file 1. After testing the calcium phosphate transfection method and several commercially available lipid based formulations for transfection of salmon kidney cells without ever exceeding 10% efficiency, we decided to evaluate electroporation as an alternative. As our main long-term goal was to establish stably transfected cell lines from Atlantic salmon we first employed the pFRT plasmid containing Flp recombination target sequences that allow subsequent integration of the gene of interest using the Flp-In vector systems. To assess the potential transfection efficiency of salmonid cell lines, the pFRT-GFP-Zeo plasmid was transfected into TO cells by electroporation using a range of buffers and programs. Three different buffers in combination with eight different settings (electric pulse programs) were tested. This optimization kit contains enough material for one round of transfections. Directly after transfection, cell viability was assessed by trypan blue exclusion assay. Viability ranged from 72 to 100% with lowest mortality using buffer T (9%, not shown). Figure 1 and 2 (respectively) display cell viability (light diffraction) and EGFP expression (fluorescence) 3 days later. A combination of buffer T with pulse program 20 or 27 was optimal for both cell integrity and frequency of GFP expression (no significant difference). As pulse program T-20 gave higher viability directly after transfection, we used this program in subsequent experiments.

### Effect of plasmid concentration

Based on published reports [20,21] and own experience during the course of the project, we learned that CMV was the most efficient promoter to drive expression of a plasmid in fish cells. In subsequent experiments we therefore used plasmids with this promoter. As plasmid concentration is important for obtaining high transfection efficiency, we wanted to determine the plasmid concentration that would give the highest transfection efficiency. Concentrations from 2.5-10 μg of the pmaxFP-Green plasmid was transfected into ASK and TO cells by electroporation using the buffer T and program T-20. Maximal transfection efficiency was achieved after 48 h and remained stable for at least one week. In ASK cells, frequency could be increased from about 40 to 50% by increasing DNA concentration from 2.5 μg to 5 or 10 μg, whereas in TO cells maximal transfection was achieved with 2.5 μg DNA.

### Sensitivity to antibiotics

**Figure 3:**
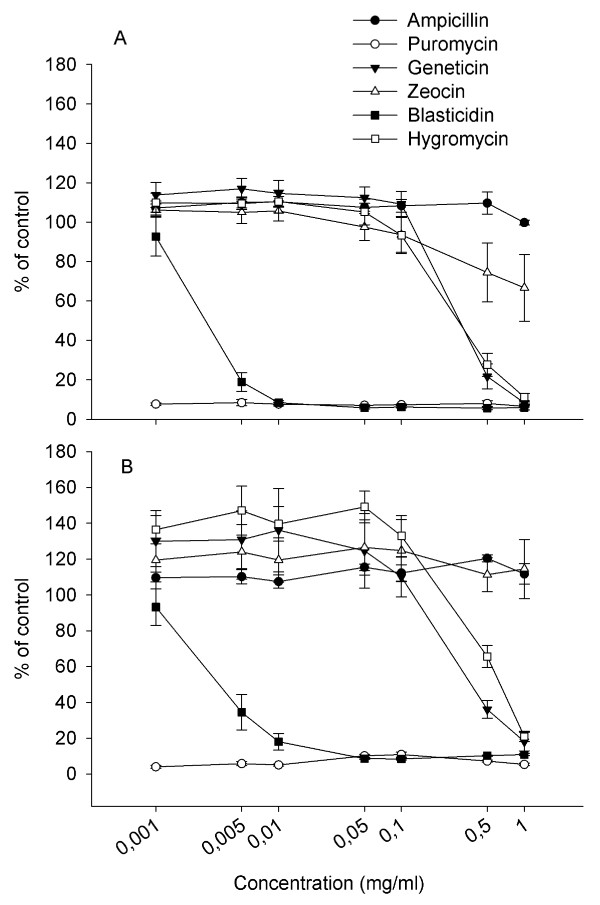
**Antibiotic sensitivity curve for SHK-1 (a) and TO (b) cells**. Cells were seeded at a density of 25 000 cells/well and incubated with a range of concentrations of the antibiotics tested. After two weeks, cell viability was measured using the cyquant cell proliferation assay. Data were normalized to cells in normal medium (100% viability) and expressed as mean of 3 experiments ± SE.

In order to determine the efficiency of selection media on TO and SHK-1, cell cultures were treated with the six different antibiotics most commonly used for selection of eukaryotic cells. Results shown in Figure 3a (SHK-1) and b (TO) shows that the salmon cell lines are most sensitive to puromycin and blasticidin. These drugs were effectively killing salmon cells at concentrations above 0.01 mg/ml whereas the other antibiotics tested were only effective above 0.5-1 mg/ml or inactive. Therefore, blasticidin and puromycin are probably well-suited antibiotics for rapid selection of stable cell lines from Atlantic salmon. A suitable vector expressing blasticidin resistance, pSELECT-blasti-mcs, was therefore chosen for stable transfection studies.

**Figure 4:**
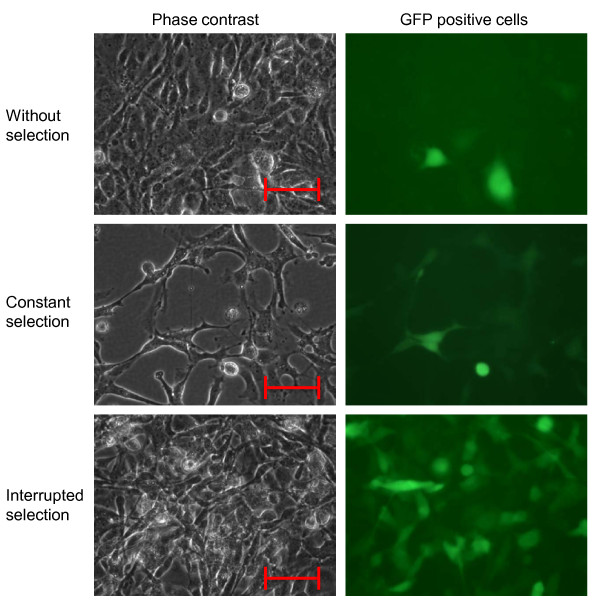
**Phase contrast and fluorescent micrographs (40X objective) of GFP positive TO cells after growth in normal medium (upper panels) and after constant selection with 0.05 mg/ml blasticidin (middle panels) or after selection with 0.05 mg/ml blasticidin for 2 weeks and then normal medium for 1 week (lower panels)**. Scale bar = 50 um.

Blasticidin resistant and GFP-expressing cells were obtained by transfection with the pSELECT-blasti-mcs plasmid followed by selection. In cultures not selected with blasticidin, the culture was dominated by cells not expressing GFP (Figure 4, upper panel). In cultures selected for 3 weeks with 0.05 mg/ml blasticidin, many cells were GFP positive, but poor growth was observed (Figure 4, middle panel). However, in cultures where selective medium was replaced with normal medium for one week, growth was restored and the fraction of GFP positive cells was further increased (Figure 4, lower panel). When cells were selected with 0.01 mg/ml blasticidin, a lower fraction of GFP positive cells were observed (not shown).

## Discussion

The ability to express endogenous or foreign proteins in cell cultures has made an important impact in modern experimental biology. This technique is used to investigate protein localization and function in health and disease. Expression of mutant proteins has greatly facilitated the understanding of many heritable human diseases. In virology, the ability to express viral proteins one by one in the absence of infection has been important for the dissection of their functions during the replication cycle. This method has not been exploited fully in the study of marine viruses, partly because of rather inefficient means to introduce and express the plasmids in cells from marine organisms, often cultured at low temperatures. We have therefore investigated various transfection methods using salmon cell lines grown at 20°C. In this report we have shown that, the nucleofector technology can be used to increase transfection efficiency up to 90%, depending on cell type. This is a significant improvement compared to our own experience with transfection of fish cells and published reports using other methods [5,6,22,23]. In contrast to lipid-based transfection, where DNA is transferred into the cytoplasm and can only enter the nucleus during mitosis, when the nuclear envelope is disintegrated, nucleofection is supposed to deliver DNA into the nucleus independent of cell cycle. High gene expression is dependent on the efficiency of the promoter under which the gene of interest is expressed. Even though the SV40 (Simian virus 40) promoter has not been shown to be efficient in fish cells [24], we wanted to use the Flp-In system to create a stable host cell line, as this would ease later introduction of genes of interest. However, it was not possible to obtain good transfection efficiency, probably due to a suboptimal promoter. The vector has zeocin resistance, and as our study shows, zeocin is not a potent drug for killing of untransfected Atlantic salmon cells. In the present study we also present data on how to successfully perform selection in Atlantic salmon cell culture. For selection purposes, the promoter driving the expression of the antibiotic resistant marker is also important. When using the vector pSELECT-blasti-mcs, with blasticidin resistance driven by the CMV promoter and the hEF1-HTLV promoter in front of GFP, we were able to obtain cell culture consisting of blasticidin-resistant, GFP positive cells. Compared to antibiotics traditionally used for efficient selection in mammalian systems, we show that blasticidin is a potent antibiotic for selection in cell lines from Atlantic salmon. Blasticidin is reported as a potent antibiotic in mammalian cell lines [25]. It works by inhibiting amino acid incorporation into polypeptides. Use of blasticidin or puromycin can significantly increase success in establishing stable cell lines. Evidently, of the antibiotics tested here, nucleoside antibiotics seem to kill non-resistant cells most efficiently. Another aspect concerning selection for stable cell lines is the amount of antibiotics used. Using an antibiotic causing rapid death of non-resistant cells at low concentrations is cost-efficient. Stable fish cell lines from other species have been reported [10,12,26,27] and further studies are in progress to establish stable cell lines from Atlantic salmon.

Establishing a protocol for transfection, we also looked into the effect of the amount of DNA on transfection efficiency. The results indicate that the protocol is optimized for TO cells, as varying the amount of DNA has little effect on transfection efficiency. For ASK cells, the transfection efficiency increases when plasmid DNA concentration is increased from 2.5 - 5 μg. Increasing the amount of DNA from 5 - 10 μg, did not seem to have an effect. The much lower efficiency observed for ASK cells, indicates that further optimization, e.g. with nucleofection- buffer or program, is needed to increase transfection efficiency.

There are many reports on successful nucleofection of mammalian primary cells and cell lines, and also a report on successful nucleofection of primary zebrafish fibroblasts [28]. Our findings have been communicated in meetings and been implemented by other groups [15,16,29]. The transfection efficiency reported here is sufficient for biochemical studies and will hopefully also be an important contribution to the regeneration of virus from plasmids (reverse genetics) or studies involving siRNA in Atlantic salmon cell lines. Although not tested here, there are reasons to believe that plasmid size will affect transfection efficiency. In a study by Kreiss et al, it was demonstrated that a 2.9 kb plasmid containing a luciferase gene was expressed between 6 and 77 times higher than a 52.5 kb plasmid containing the same gene, dependent on cell type [30]. The plasmids used here were between 3.9 kb and 5.7 kb and therefore in the lower range.

## Conclusions

In summary, we demonstrate improved transfection of cell lines from Atlantic salmon using nucleofection; we also propose a method for selection of transfected cells in selective medium containing blasticidin. This is of immediate interest for studies involving transient transfection of the cell lines used and may be of importance for future establishment of foreign or recombinant protein-expressing cell lines from Atlantic salmon.

## Competing interests

The authors declare that they have no competing interests.

## Authors' contributions

BLS designed the conducted the experiments with the antibiotic toxicity and -selection, designed the pSELECT-GFP-blasti-mcs plasmid and wrote and finalized the manuscript. EGR and HS designed and conducted the experiment with the optimization of electroporation conditions. ESB designed and conducted the experiment with optimization of DNA concentration and GFP expression over time. ML constructed the pFRT-GFP-Zeo plasmid. SM designed the experiments with optimization of electroporation conditions and DNA concentration and GFP expression over time, and edited the manuscript. UG designed the experiments with optimization of electroporation conditions. TG designed the experiments with the antibiotic toxicity and -selection and edited the manuscript. All authors have read and approved the final manuscript.

## Supplementary Material

Additional file 1 **Supplementary methods**. The materials and methods from this study Click here for file
