# Supplementary material for: Enhanced transfection of cell lines from Atlantic salmon through nucoleofection and antibiotic selection
Source: BMC Res Notes. 2011 May 6;4:136. doi: 10.1186/1756-0500-4-136 (PMC3113957; doi:10.1186/1756-0500-4-136)
Supplement: Additional file 1 — Supplementary methods. The materials and methods from this study [file 1756-0500-4-136-S1.DOCX]

Additional file 1

Supplementary methods

## Reagents

Cell Line Nucleofector Solutions R, T, V and supplement were from Amaxa Biosystems (Cologne, Germany). These solutions are optimized for different cell types but their exact content is proprietary information. FuGene 6 Transfection reagent was from Roche (Roche Diagnostics GmbH, Mannheim, Germany). Leibovitz’s L-15 medium, gentamicin and L-glutamine was from Cambrex Bio Sciences (Cambrex Bio Sciences, Verviers, Belgium). Trypsin-EDTA, β-mercaptoethanol and fetal calf serum was from Gibco-Invitrogen (Paisley, UK). Ampicillin, geneticin, hygromycin and puromycin were from Sigma-Aldrich (St. Louis, MO, USA), while zeocin and blasticidin S HCl were from Invitrogen (Carlsbad, CA, USA). Restriction enzymes were from MBI Fermentas (St. Leon-Rot, Germany).

## Cell culture

TO [24], SHK-1 [25] and ASK (kindly provided by Bjorn Krossoy, Bergen, Norway) cells were cultured at 20°C in Leibovitz’s L-15 medium supplemented with 5% (TO, SHK-1) or 10% (ASK) fetal calf serum, 4mM L-glutamine, 50µg ml^-1^ gentamicin and 40 µM β-mercaptoethanol. All 3 cell lines have been isolated from head kidney of Atlantic salmon.

## Plasmids

The pmaxFP-Green-N vector, used for optimization of plasmid concentration, was from Amaxa Biosystems. It encodes green fluorescent protein, maxFP-Green under the control of the SV40 promoter and provides neomycin resistance. Its molecular size is 4.7 kb. The pFRT/lacZeo Flp vector was from Invitrogen, (Carlsbad, CA, USA). It contains FRT recombination target sequences that allow subsequent integration of the gene of interest using the Flp-In vector systems. The vector contains a LacZ-Zeocin resistance fusion gene under the control of the SV40 early promoter. The pSELECT-blasti-mcs vector was from InvivoGen (San Diego, CA, USA). It contains two expression cassettes; the first drives the expression of the gene of interest under the hEF1-HTLV promoter, comprising the Elongation Factor-1α (EF-1α) and the R segment and part of the U5 sequence (R-U5’) of the Human T-Cell Leukemia Virus (HTLV) Type 1, and the second drives the expression of selection marker blasticidin, under the control of the cytomegalovirus (CMV) immediate-early promoter. The pFRT/lacZeo Flp and pSELECT-blasti-mcs have been modified to introduce the EGFP (enhanced green fluorescent protein) gene from the pEGFP-N1 plasmid.

Construction of pFRT-GFP-Zeo

The lacZ-gene was removed by digesting the pFRT/lacZeo plasmid with HindIII and Bsp119I. GFP was amplified from pEGFP-N1 using the following primers with restriction sites (underlined) GFP_Fwd_HindIII: 5’-CAAGCTTGGCACTGGTGAGCAAGGGCGAGGAGCTGTT and GFP_Rev_Bsp119I: 5’- CTTTCGAAAGCGAATTGTACAGCTCGTCCATGCCG. The amplified product was initially cloned into pCR BluntII-TOPO. GFP was digested from pCR BluntII with HindIII and Bsp119I, and after purification from gel using QIAquick Gel Extraction Kit (QIAGEN) and ligated into pFRT-Zeo using T4 DNA ligase (Invitrogen, Carlsbad, CA, USA) at 16°C for 18 h. The total size of this plasmid was 5.7 kb.

Construction of pSELECT-GFP-blasti-mcs

Enhanced green fluorescent protein (EGFP) was amplified from the pEGFP-N1 plasmid (Clontech, Palo Alto, CA, USA) using the following primers with restriction sites (underlined) BamHI-pEGFP_593F 5’ – GGGGGGATCCTAGCGCTACCGGACTCAGAT – 3’ and NheI-pEGFP_1480R 5’ – GGGGGCTAGCGGGAGGTGTGGGAGGTTTT – 3’. The PCR product was ligated into the pGEM-T-Easy vector system. The pGEM vector was digested with BamHI and NheI and subcloned into pSELECT-blasti-mcs vector using T4 DNA ligase (Invitrogen) at 16°C for 18 h, giving a new construct of size 3.9 kb.

## Transfection

Optimization of conditions for electroporation

Cells were split three days before transfection, and 5x10^6^ cells and 2.5-10 µg plasmid DNA was used for each transfection. The cells were transfected using the Amaxa nucleofector TM, varying the buffers and Amaxa programs. The different programs subject the cells to electric pulses of varying amplitude, frequency and duration. Directly after transfection, the cells were resuspended in RPMI medium, and then transferred to supplemented L-15 medium. Cell viability directly after transfection was analyzed by dye exclusion method (0.2% trypan blue) in a haemocytometer. Three days later, cells were trypsinized and analyzed with respect to viability (light diffraction) and transfection frequency (GFP fluorescence) by flow cytometry (FACS Calibur, Becton Dickinson)

The initial transfection studies in Atlantic salmon cells using calcium phosphate or lipid-based reagents were performed according to the established protocols and manufacturers’ instructions.

## Antibiotic toxicity studies

Cells were seeded at a density of 25 000 cells/well in white 96-well microplates (Nunc, Roskilde, Denmark). The cells were allowed to attach over night. A range of antibiotics in concentrations from 0.001 mg/ml to 1 mg/ml was added to the wells. The cells were incubated at 20ºC for 2 weeks. Cytotoxicity was then measured with the Cyquant cell proliferation assay kit (Molecular Probes-Invitrogen, Eugene, OR, USA).

## Selection of GFP expressing cells

The cells were transfected with the pBlasti-select plasmid and cultured for 6 days before blasticidin was added to the medium in a concentration of 0.01 mg/ml or 0.05 mg/ml. After two weeks, the medium in some flasks were replaced by normal medium to promote good growth. Development of GFP-expressing cell colonies was observed using a fluorescence microscope.
